# The incretin hormone glucagon‐like peptide 1 increases mitral cell excitability by decreasing conductance of a voltage‐dependent potassium channel

**DOI:** 10.1113/JP272322

**Published:** 2016-04-13

**Authors:** Nicolas Thiebaud, Ida J. Llewellyn‐Smith, Fiona Gribble, Frank Reimann, Stefan Trapp, Debra Ann Fadool

**Affiliations:** ^1^ The Florida State UniversityDepartment of Biological Science, Program in NeuroscienceTallahasseeFLUSA; ^2^ Cardiovascular Medicine and Human Physiology, School of MedicineFlinders UniversityBedford ParkSAAustralia; ^3^ Cambridge Institute for Medical ResearchAddenbrooke's HospitalCambridgeUK; ^4^ Centre for Cardiovascular and Metabolic Neuroscience, Department of Neuroscience, Physiology, and PharmacologyUniversity College LondonLondonUK; ^5^ Department of Surgery and CancerImperial College LondonLondonUK; ^6^ The Florida State UniversityInstitute of Molecular BiophysicsTallahasseeFLUSA

## Abstract

**Key points:**

- The gut hormone called glucagon‐like peptide 1 (GLP‐1) is a strong moderator of energy homeostasis and communication between the peripheral organs and the brain.
- GLP‐1 signalling occurs in the brain; using a newly developed genetic reporter line of mice, we have discovered GLP‐synthesizing cells in the olfactory bulb.
- GLP‐1 increases the firing frequency of neurons (mitral cells) that encode olfactory information by decreasing activity of voltage‐dependent K channels (Kv1.3).
- Modifying GLP‐1 levels, either therapeutically or following the ingestion of food, could alter the excitability of neurons in the olfactory bulb in a nutrition or energy state‐dependent manner to influence olfactory detection or metabolic sensing.
- The results of the present study uncover a new function for an olfactory bulb neuron (deep short axon cells, Cajal cells) that could be capable of modifying mitral cell activity through the release of GLP‐1. This might be of relevance for the action of GLP‐1 mimetics now widely used in the treatment of diabetes.

**Abstract:**

The olfactory system is intricately linked with the endocrine system where it may serve as a detector of the internal metabolic state or energy homeostasis in addition to its classical function as a sensor of external olfactory information. The recent development of transgenic mGLU‐yellow fluorescent protein mice that express a genetic reporter under the control of the preproglucagon reporter suggested the presence of the gut hormone, glucagon‐like peptide (GLP‐1), in deep short axon cells (Cajal cells) of the olfactory bulb and its neuromodulatory effect on mitral cell (MC) first‐order neurons. A MC target for the peptide was determined using GLP‐1 receptor binding assays, immunocytochemistry for the receptor and injection of fluorescence‐labelled GLP‐1 analogue exendin‐4. Using patch clamp recording of olfactory bulb slices in the whole‐cell configuration, we report that GLP‐1 and its stable analogue exendin‐4 increase the action potential firing frequency of MCs by decreasing the interburst interval rather than modifying the action potential shape, train length or interspike interval. GLP‐1 decreases Kv1.3 channel contribution to outward currents in voltage clamp recordings as determined by pharmacological blockade of Kv1.3 or utilizing mice with Kv1.3 gene‐targeted deletion as a negative control. Because fluctuations in GLP‐1 concentrations monitored by the olfactory bulb can modify the firing frequency of MCs, olfactory coding could change depending upon nutritional or physiological state. As a regulator of neuronal activity, GLP‐1 or its analogue may comprise a new metabolic factor with a potential therapeutic target in the olfactory bulb (i.e. via intranasal delivery) for controlling an imbalance in energy homeostasis.

## Key points

- The gut hormone called glucagon‐like peptide 1 (GLP‐1) is a strong moderator of energy homeostasis and communication between the peripheral organs and the brain.
- GLP‐1 signalling occurs in the brain; using a newly developed genetic reporter line of mice, we have discovered GLP‐synthesizing cells in the olfactory bulb.
- GLP‐1 increases the firing frequency of neurons (mitral cells) that encode olfactory information by decreasing activity of voltage‐dependent K channels (Kv1.3).
- Modifying GLP‐1 levels, either therapeutically or following the ingestion of food, could alter the excitability of neurons in the olfactory bulb in a nutrition or energy state‐dependent manner to influence olfactory detection or metabolic sensing.
- The results of the present study uncover a new function for an olfactory bulb neuron (deep short axon cells, Cajal cells) that could be capable of modifying mitral cell activity through the release of GLP‐1. This might be of relevance for the action of GLP‐1 mimetics now widely used in the treatment of diabetes.

**Abbreviations**
aCSF: artificial cerebral spinal fluid
AP: action potential
AVMA: American Veterinary Medicine Association
ASPA: Animals Scientific Procedures Act
DAPI: 4′,6‐diamidino‐2‐phenylindole
dSAC: deep short axon cell
Ex4: exendin‐4
EPL: external plexiform layer
GLP‐1: glucagon‐like peptide‐1
GLP‐1R: glucagon‐like peptide 1 receptor
GCL: granule cell layer
GFP: green fluorescent protein
ISI: interspike interval
IACUC: Institutional Animal Care and Use Committee
Kv1.3^−/−^: Kv1.3 gene‐targeted deletion
MGTX: margatoxin
MC: mitral cell
MCL: mitral cell layer
OB: olfactory bulb
OMP: olfactory marker protein
PBS: phosphate‐buffered saline
PFA: paraformaldehyde
PPG: preproglucagon
RMP: resting membrane potential
WT: wild‐type
YFP: yellow fluorescent protein

## Introduction

Energy homeostasis is dependent upon neuronal and hormonal communication between the peripheral organs and the brain. Glucagon‐like peptide 1 (GLP‐1) is an incretin hormone secreted from intestinal L‐cells following a meal (Holst, 2007). Peripheral GLP‐1 exerts strong effects on the control of energy metabolism by regulating glycaemia, stimulating glucose‐dependent insulin secretion and proinsulin gene expression, and inhibiting glucagon release and gastric emptying (Holst, 2007). In the central nervous system, cell bodies of GLP‐1 producing neurons have been identified in the caudal nucleus of the solitary tract and intermediate reticular nucleus (Jin *et al*. 1988; Larsen *et al*. 1997; Merchenthaler *et al*. 1999; Hisadome *et al*. 2010; Trapp & Richards, 2013). These neurons send axons to several areas in the brain, including the paraventricular nucleus, the dorsomedial hypothalamus and the nucleus accumbens (Larsen *et al*. 1997; Tauchi *et al*. 2008; Llewellyn‐Smith *et al*. 2011). Direct central infusion of GLP‐1 has been shown to inhibit food and water intake and to reduce blood glucose (Tang‐Christensen *et al*. 1996; Kinzig *et al*. 2002; Dossat *et al*. 2011).

The receptor for GLP‐1 (GLP‐1R) is a seven transmembrane domain, G‐protein coupled receptor that is expressed in a wide variety of tissues, including the hypothalamus and the nucleus accumbens, where GLP‐1 has been demonstrated to play a role in the control of food intake (Dossat *et al*. 2011). Ligand binding of the peptide to GLP‐1R triggers activation of the Gα_s_ protein, which leads to the production of cAMP through the activation of the enzyme adenylate cyclase. Elevation of cAMP concentration in the cell increases protein kinase A activity, which results in the inhibition of potassium channels, K_ATP_, Kv2.1 and Kv1.5, as well as the auxiliary Kvβ2 protein subunit (Gromada *et al*. 1998 *a*; Gromada *et al*. 1998 *b*; MacDonald *et al*. 2003; Kim *et al*. 2012; Kim *et al*. 2013) by phosphorylation or acetylation. This results in a reduction of the Kv current amplitude or reduced Kv surface trafficking, either of which would cause increased depolarization. In pancreatic β‐cells, GLP‐1R activation also induces an elevation of intracellular Ca^2+^ concentrations through modification of L‐type Ca^2+^ channels and the oscillation of Ca^2+^ through Ca^2+^‐induced Ca^2+^ release from intracellular stores (Holst, 2007).

Most studies investigating the second messengers involved in GLP‐1 signalling have been performed in pancreatic β‐cells where GLP‐1 stimulates glucose‐dependent insulin release. GLP‐1, however, has also been shown to increase membrane excitability of nodose ganglion neurons by decreasing the potassium conductance of both inactivating and delayed‐rectifier potassium currents (Gaisano *et al*. 2010). Nodose neurons are no longer modulated by the peptide following blockade of K^+^ currents with the relatively non‐selective antagonist, 4‐AP.

Because the olfactory system is intricately linked with the endocrine system, it may not only serve as an external sensor of olfactory information, but also as an internal sensor of metabolic state or energy homeostasis (Palouzier‐Paulignan *et al*. 2012). It has been reported previously that hormonal and metabolic factors modulate the excitability of olfactory sensory neurons, as well as mitral cells (MCs) representing the first‐order neurons in the olfactory bulb (OB) (Fadool *et al*. 2000; Fadool *et al*. 2011; Aime *et al*. 2012; Soria‐Gomez *et al*. 2014). Initial electrophysiological studies exploring neuroendocrine modulation focused upon the hormone insulin, which has the highest binding affinity and receptor density reported for the OB compared to all other brain regions (Hill *et al*. 1986; Banks *et al*. 1999). Other factors, such as orexins, have been shown to modulate MC firing frequency (Apelbaum *et al*. 2005; Hardy *et al*. 2005). Orexins originate from neurons in the lateral hypothalamic area that send centrifugal projections to the OB (Gascuel *et al*. 2012). Prud'homme *et al*. (2009) have demonstrated that the release of the orexigenic peptide within the OB is highly dependent upon nutritional status (fasted *vs*. satiated). Recently, our group demonstrated that other metabolic factors, including glucose itself, modulate the firing frequency of MCs (Tucker *et al*. 2013), suggesting that there is a glucose‐sensitive population of MCs detecting changes in glucose homeostasis. In MCs, glucose and insulin use a mechanism that involves changes in excitability driven by a voltage‐dependent potassium channel, Kv1.3, as demonstrated by the lack of response to these metabolic factors in MCs from mice with gene‐targeted deletion of Kv1.3 (Kv1.3^−/−^) (Fadool *et al*. 2011; Tucker *et al*. 2013).

Hormonal regulation occurring within the olfactory system is hypothesized to modulate olfactory sensitivity and the hedonic representation of odours following changes in nutritional state. For example, the endocanabinoid system has recently been shown to be involved in the modulation of olfactory sensitivity and food intake through activation of CB1 receptors in the olfactory system (Soria‐Gomez *et al*. 2014). Moreover, dysregulation of energy metabolism affects the olfactory system. Rodent models of genetic‐ or diet‐induced obesity have demonstrated both anatomical and functional changes in the olfactory system (Badonnel *et al*. 2012; Tucker *et al*. 2012; Thiebaud *et al*. 2014). Such changes have an impact on olfactory‐linked food behaviours or food intake.

Although a single previous study has used *in situ* hybridization to characterize the expression of preproglucagon (PPG) and the GLP‐1 receptor in the olfactory system (Merchenthaler *et al*. 1999), the physiological functions of the GLP‐1 pathway, as well as protein expression and localization, remain completely unexplored. The recent development of transgenic mGLU‐yellow fluorescent protein (YFP) mice, expressing the yellow fluorescent protein, Venus, under the control of the glucagon promoter (Reimann *et al*. 2008), allowed us to confirm the presence of GLP‐1 producing neurons in the OB. Following GLP‐1 receptor binding assays and labelling experiments in mice with a genetic reporter for MCs, we conjectured that MCs were a target for the endogenous peptide. Accordingly, we performed a biophysical analysis of GLP‐1 modulation on these first‐order neurons. Using the whole‐cell configuration of the patch clamp in OB slices, we determined the mechanism of action of GLP‐1 or its stable analogue exendin‐4 (Ex4) on MCs. Fluctuations in GLP‐1 concentrations were able to modify the intrinsic firing frequency of MCs and alter the olfactory coding in response to a changed physiological state, such as following a meal or after fasting.

## Methods

### Ethical approval

All experiments described in the present study were approved by the Florida State University Institutional Animal Care and Use Committee (IACUC) under protocols #1124 and #1427, and were conducted in accordance with the American Veterinary Medicine Association (AVMA), the National Institutes of Health, and the UK Home Office Regulations under the Animals Scientific Procedures Act (ASPA) 1986. In preparation for OB slice electrophysiology, mice were anaesthetized with isoflurane (Aerrane; Baxter, Deerfield, IL, USA) using the IACUC‐approved drop method and then were killed by decapitation (AVMA Guidelines on Euthanasia, June 2007). These procedures are contained in the ASPA Schedule 1 (Wolfenshon & Lloyd, 2003) for the species, stage of development, and size of our mice (see below). All authors understood the ethical principles that *The Journal of Physiology* operates under and the work complied with the animal ethics checklist reported by Grundy (2015).

### Animal care and mouse lines

All mice were housed at the Florida State University, Imperial College London, or University College London vivaria in accordance with the institutional requirements for animal care. All mice used in the present study (*Mus musculus*, C57BL/6 background strain; Jax Laboratories, Bar Harbor, ME, USA) were maintained under a standard 12:12 h light/dark cycle and were allowed *ad libitum* access to 5001 Purina Chow (Purina, Richmond, VA, USA) and water. Mice of both sexes were aged from postnatal day 21 to 35 at the time of the experiment unless otherwise noted, and had a body weight in the range 8.4–20.0 g (mean ± SD: 15.9 ± 2.5 g). A total of 62 wild‐type (WT), 12 Kv1.3^−/−^, three olfactory marker protein‐green fluorescent protein (OMP‐GFP), nine PPG‐YFP, three GLP‐1R‐tdRFP, three Thy1‐YFP and three GLP‐1R^−/−^ mice were used in the present study. Kv1.3^−/−^ mice were produced previously by excision of the Kv1.3 promoter region and one‐third of the 5′ coding region (Koni *et al*. 2003; Xu *et al*. 2003) and were a generous gift from Drs Leonard Kaczmarek and Richard Flavel (Yale University, New Haven, CT, USA). Mice with an olfactory marker protein GFP transgene (OMP‐GFP) (Potter *et al*. 2001) were used to identify all mature olfactory sensory neurons and were a gift from Dr Peter Mombaerts (Max Plank Institute, Frankfort, Germany). PPG‐YFP mice, expressing the YFP variant Venus (Nagai *et al*. 2002) under the control of the mouse PPG promotor (mGLU‐124 line) (Reimann *et al*. 2008), were used to identify proglucagon expressing neurons. Briefly, mice were generated using mouse bacterial artificial chromosomes using the RedEt technique (Zhang *et al*. 2000). Pronuclear injection of bacterial artificial chromosome constructs containing the Venus sequence in place of the coding region of PPG resulted in the generation of founder mice with the transgene (mGLU‐124 line). GLP‐1R Cre mice that express Cre‐recombinase under the GLP‐1R promoter were generated as described previously (Richards *et al*. 2014). These mice were crossed with ROSA26‐tdRFP reporter strains (Luche *et al*. 2007) to enable fluorescence detection of cells expressing GLP‐1R by cytosolic tdRFP expression. Thy1‐YFP mice were used to identify MCs and were a gift from Dr Guoping Feng (MIT Broad Institute, Boston, MA, USA) (Feng *et al*. 2000). GLP‐1R^−/−^ mice were a generous gift from Dr Julio E. Ayala (Diabetes and Obesity Research Centre, Orlando, FL, USA) (Ayala *et al*. 2009).

### Solutions, reagents and antisera

Margatoxin (MGTX) was purchased from Sigma‐Aldrich (St Louis, MO, USA) and used to selectively block the vestibule of the Kv1.3 channel (Knaus *et al*. 1995). TTX was purchased from Ascent Scientific (Princeton, NJ, USA) or Abcam Biochemicals (Cambridge, MA, USA) to block voltage‐gated sodium channels. GLP‐1, glucagon‐like peptide 1 (7‐36)‐lys (biotin) (Ref 46‐1‐65) and GLP‐1‐(7‐36) amide were purchased from American Peptide Company (Sunnyvale, CA, USA). Phosphate‐buffered saline (PBS) was made as described previously (Tucker & Fadool, 2002). Slice intracellular pipette solution contained (in mm): 135 potassium gluconate, 10 KCl, 10 Hepes, 10 MgCl_2_, 0.4 NaGTP and 2 NaATP (pH 7.3; 280–285 mOsm). Artificial cerebral spinal fluid (aCSF) contained (in mm): 119 NaCl, 26.2 NaHCO_3_, 1 NaH_2_PO_4_, 2.5 KCl, 1.3 MgCl_2_, 2.5 CaCl_2_ and 22 d‐glucose (pH 7.3; 310–315 mOsm). In experiments where the external concentration of potassium was adjusted to alter the calculated reversal potential for K^+^, KCl was modified to 2.5 or 8.5 mm. Sucrose‐modified aCSF was used for sectioning and contained (in mm): 83 NaCl, 26.2 NaHCO_3_, 1 NaH_2_PO_4_, 3.3 MgCl_2_, 0.5 CaCl_2_, 72 sucrose and 22 d‐glucose (pH 7.3; 315 mOsm) (De Saint Jan & Westbrook, 2007). All salts and sugars were purchased from Sigma‐Aldrich or Fisher Scientific (Pittsburgh, PA, USA).

The GLP‐1R antibody (dilution 1:3000) was a generous gift from Dr Joel Habener (Harvard Catalyst, Cambridge, MA, USA) and Dr Scott Heller (Hagedorn Research Institute, Denmark) and was generated against the N‐terminal epitope (CQHRYERWKQVTESLSVT) in rabbits (Heller *et al*. 1996). Chicken anti‐GFP was purchased from Abcam Biochemicals (dilution 1:50,000, catalogue number ab13970) and was designed to recognize the enhanced form of GFP, and all of the fluorescent proteins made by *Aequorea victoria*, including YFP. The insulin receptor (IR) kinase antibody was a mouse monoclonal directed against the β subunit (dilution 1:1000, Clone CT‐3; catalogue number 05‐1104; Millipore, Billerica, MA, USA). Host‐specific secondary antibodies were purchased from Jackson ImmunoResearch (West Grove, PA, USA) and used in accordance with the manufacturer's instructions. GLP1‐R expressing cells from GLP‐1R transgenic line were detected using a far red polyclonal antibody, DsRed (dilution 1:1000, catalogue number 632496; Clontech, Palo Alto, CA, USA) using protocols described previously (Cork *et al*. 2015).

### Immunocytochemistry

YFP‐immunoreactivity in GLP‐1 neurons was visualized in PPG‐YFP mice as described previously (Llewellyn‐Smith *et al*. 2011; Llewellyn‐Smith *et al*. 2013). Briefly, a total of five mice aged 12–16 weeks and weighing between 25 and 35 g were perfused intracardially with 4% paraformaldehyde (PFA). The head was subsequently postfixed in 4% PFA/PBS overnight and decalcified for 3–5 days in 0.3 m EDTA and prepared for cryosectioning (30 μm) as described in Thiebaud *et al*. (2014). YFP‐immunoreactivity was confirmed in parallel experiments using an antibody to GFP (see antibodies). Here, an immunoperoxidase protocol in conjunction with a metal‐intensified diaminobenzidine reaction was used to optimally visualize the morphology of GLP‐1 neurons (Llewellyn‐Smith *et al*. 2011). A total of four mice for the immunoperioxidase studies were perfused with 4% PFA/PBS. Brains with attached OBs were removed and post‐fixed for 3 days at room temperature in the same fixative. OBs were infiltrated with sucrose and cryosectioned (30 μm). A BH‐2 microscope (Olympus, Tokyo, Japan) equipped with a SPOT Insight Model 18.2 firewire colour camera and SPOT, version 4.6 (Diagnostic Instruments, Inc., Sterling Heights, MI, USA) was used to capture images.

To localize GLP‐1R by immunocytochemical approaches, a total of three adult mice were used. Mice were intracardially perfused with 4% paraformaldehyde in PBS (PFA/PBS). The OBs were postfixed overnight in 4% PFA, decalcified for 3–5 days in 0.3 m EDTA, and then prepared for cryosectioning (12 μm) as described in Thiebaud *et al*. (2014). Prepared OB frozen sections were air‐dried for 30 min, fixed in 1% PFA/PBS, washed three times in PBS and then incubated for 30 min in 3% bovine serum albumin/PBS to block non‐specific binding. For use of the IR kinase antibody, it was also necessary to perform a demasking step by boiling sections (i.e. microwave) in 10 mm citrate buffer (pH 6.0) for 4 min for epitope retrieval prior to the blocking step in accordance with the manufacturer's instructions. After the blocking step, the tissue sections were incubated overnight with the primary antiserum (dilution in PBS + 3% BSA) at the same time as protecting from light and maintaining sections at 4°C. The immunofluorescence signal was detected after a 2 h incubation with host‐specific Cy3 or Cy2 secondary antisera diluted in PBS (dilution 1:200; Jackson ImmunoResearch). After three washes in PBS, nuclei were additionally stained by incubating slides for 5 min in 4′,6‐diamidino‐2‐phenylindole (DAPI) nuclear stain diluted in PBS (dilution 1:15,000; Life Technologies, Carlsbad, CA, USA), washing again in PBS and coverslipping with Fluoromount G (Southern Biotechnology, Birmingham, AL, USA) to prevent photobleaching. OB sections were examined by brightfield and fluorescence using an Axiovert S100 inverted microscope (Carl Zeiss Microimaging, Thornwood, NY, USA). Digital images were captured with an Axiocam digital camera and AxioVision, version 4.8 (Carl Zeiss Microimaging).

### GLP‐1 binding assay

The GLP‐1‐biotin binding assay was performed on three WT mice as adapted from Cowley *et al*. 2003. Briefly, 150 μm vibratome sections of OB were obtained in ice‐cold, sucrose‐modified aCSF. The oxygenated (95% O_2_/5% CO_2_) sections recovered for 1 h in an interface chamber in normal aCSF (Fadool *et al*. 2011) and were then incubated with either 1 μm GLP‐1‐biotin conjugate or a mix of 1 μm GLP‐1‐biotin conjugate with 2 μm of ‘cold’ GLP‐1 (unconjugated) for 20 min at +4ºC. The sections were subsequently fixed with 4% PFA/PBS for 10 min, washed in PBS and incubated in 1:200 Cy3‐streptavidin (Life Technologies) for 30 min. The sections were stained with DAPI nuclear stain (dilution 1:15,000), washed in PBS, and then mounted and visualized as described above.

In another experiment, Thy‐1‐YFP mice that express YFP in MCs or GLP‐1R^−/−^ mice received a s.c. injection (120 nmol kg^−1^) of fluorescence‐labelled Ex4 [Cys(HiLyte Fluor647 C2 maleimide)]‐exendin‐4 (Ref AS‐63714; AnaSpec, Fremont, CA, USA). The animals were transcardially perfused with 4% PFA 4 h after injection and the OBs were processed and cryosectioned at a thickness of 12 μm as described previously (Thiebaud *et al*. 2014).

### OB slice electrophysiology

A total of 79 mice were used for 197 patch clamp electrophysiology recordings from OB slices as described previously (Fadool *et al*. 2011). Following isoflurane anaesthesia, the mice were killed by decapitation. The OBs were rapidly exposed by removing the dorsal and lateral portions of the cranium between the cribriform plate and the lambda suture as described previously (Nickell *et al*. 1996). After removing the dura, the OBs (when still attached to the forebrain) were quickly removed, glued to a sectioning block with Superglue (Lowe's Home Improvement, Tallahassee, FL, USA) and submerged in oxygenated, ice‐cold, sucrose‐modified aCSF to prepare the tissue for sectioning (De Saint Jan & Westbrook, 2007). Coronal sections (275 μm) were cut in oxygenated, ice‐cold, sucrose‐modified aCSF using a Series 1000 Vibratome (Leica, Wetzlar, Germany). The sections were allowed to recover in an interface chamber (Krimer & Goldman‐Rakic, 1997) with oxygenated, sucrose‐modified aCSF at 33°C for 30 min and then maintained at room temperature in oxygenated normal aCSF until needed (De Saint Jan & Westbrook, 2007; Fadool *et al*. 2011). Neuronal slices were visualized at 10× and 40× using an Axioskop 2FS Plus microscope (Carl Zeiss Microimaging) equipped with infrared detection capabilities (CCD100; Dage‐MTI, Michigan City, IN, USA).

**Figure 1:**
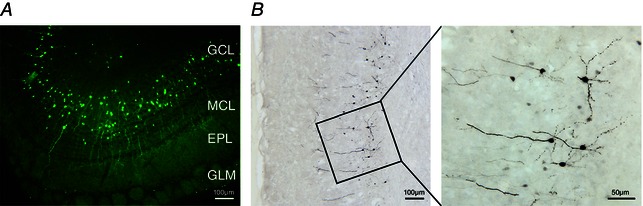
**Preproglucagon positive (PPG+) short axon cells are visualized in the OB** *A*, photomicrograph of a representative coronal section of the mouse OB of a PPG‐YFP mouse demonstrating native YFP labelling of dSAC within the GCL. Also visible in the section are other neurolamina, MCL, EPL and the glomerular cell layer (GLM). Note the position of the soma in the upper portion of the GCL and the axon projections into the MCL and EPL. *B*, same as in (*A*) but labelled using a metal‐intensified diaminobenzidine reaction in conjunction with an anti‐GFP antibody to optimally visualize the short axon morphology. Inset: higher magnification of the boxed area is shown on the right. Note that the YFP‐immunoreactive cells are stellate with dendrites containing spine‐like structures or a varicose appearance.

Membrane voltage and current properties were generated using pCLAMP, version 9 or 10, in conjunction with an Axopatch 200B amplifier (Axon Instruments, Foster City, CA, USA). The analogue signal was filtered at 2 kHz and minimally digitally sampled every 100 μs. Electrodes were fabricated from borosilicate glass (#1405002; Hilgenberg GmbH, Malsfeld, Germany) to a diameter of ∼2 μm to yield pipette resistances ranging from 4 to 7 MΩ. Positive pressure was retained when navigating through the OB laminae until a high resistance seal (1.5–10 GΩ) was obtained on a positionally‐identified MC in the slice (Fadool *et al*. 2011). The morphology and biophysical properties of the neurons were used to distinguish MCs from tufted cells. In addition, thy1‐YFP transgenic mice comprised a good tool to secondarily confirm cell identity as in the study by Fadool *et al*. 2011 (Fig. 1). The whole‐cell configuration was established by applying gentle suction to the lumen of the pipette at the same time as monitoring resistance. Each MC was first sampled for adequate resting potential (less than –55 mV) and proper series resistance (less than 40 MΩ) prior to initiating a series of current clamp recordings. Only current clamp recordings with a sustained resting membrane potential (RMP) of at least –50 mV or an input resistance (*R*_in_) of at least 150 MΩ were accepted. During recordings, the membrane potential (*E*_m_) was adjusted to –65 mV by injecting a few pA of current so that data could be statistically evaluated from the same potential across cells.

For current clamp recordings, the perithreshold current level was determined by incrementally injecting 1 s long, 25 pA steps of current every 10 s, starting at –50 pA. Following the determination of spike threshold, cells were then stimulated with a long, perithreshold current step (typically ranging from 25 to 100 pA) of 5000 ms at an interpulse interval of 10 s to acquire spike frequency data before and after peptide or drug application. In general, recordings were acquired for a minimum of 30 min following application of peptide. Latency to first spike, spike event frequency (calculated throughout step depolarization), interburst interval (calculated between spike cluster), interspike interval (ISI) (calculated within a spike cluster) and action potential (AP) cluster length were measured as described previously (Balu *et al*. 2004; Fadool *et al*. 2011; Mast & Fadool, 2012). A cluster was defined as three or more consecutive spikes with an ISI of 100 ms or less as established by Balu *et al*. (2004). Because MC firing is intrinsically intermittent and is characterized by variable spike clusters, classical means of computing spike timing variability, such as peristimulus time histograms, were less suitable for the behaviour of these neurons and therefore alternative means of spike analysis were applied as described previously (Balu *et al*. 2004).

For voltage clamp recordings, voltage‐activated outward currents were elicited using a 400 ms voltage stimulation protocol with a holding potential (*V*_h_) of –80 mV and command potential (*V*_c_) steps from –100 to +40 mV in 10 mV increments. Interpulse intervals were taken at 45 s to prevent cumulative inactivation of the Kv1.3 current that is expressed in MCs (Marom *et al*. 1993; Fadool & Levitan, 1998).

### Statistical analysis

All electrophysiological data were analysed using pClamp, version 10 (Clampfit 10.2; Axon Instruments), Prism, version 5 (GraphPad Software, Inc., La Jolla, CA, USA) and Igor Pro, version 6.12A (WaveMetrics Inc., Portland, OR, USA) with the plug‐in NeuroMatic, version 2 (written by Jason Rothman; http://www.neuromatic.thinkrandom.com). The pipette capacitance was electrically compensated through the capacitance neutralization circuit of the Axopatch 200B amplifier. Similarly, series resistance compensation was used to electrically reduce the effect of pipette resistance. Voltage clamp traces were subtracted linearly for leakage conductance. Resting membrane potentials were corrected for a –14 mV junction potential offset.

For voltage clamp experiments, the peak transient current was defined as the greatest current evoked after voltage activation. Current–voltage relationships were plotted by normalizing the peak transient current to either cell capacitance or to the maximum evoked current at +40 mV. The family of current–voltage relations was compared (via Prism) using a repeated measures two‐way ANOVA (and a Bonferoni *post hoc* test) with peptide and voltage step as factors. For current clamp experiments, change in the AP frequency, ISI or interburst interval were plotted as the mean percentage change compared to the control condition prior to bath application of the peptide. Baseline, treatment and washout values were calculated from the mean of 10 consecutives traces generated from a 5 s duration current injection. For statistical analyses, Prism was used to perform the Friedman test (non‐parametric alternative for repeated measures ANOVA) followed by Bonferoni's *post hoc* comparison test. All data are reported as the mean ± SEM. Different letters denote statistically different mean values with the corresponding *P* value as specified. Statistical difference was defined at the 95% confidence interval (or α ≤ 0.05), unless otherwise specified.

## Results

### Localization of PPG‐neurons in the OB

Immunocytochemical localization of GLP‐1 peptide can yield precarious results as a result of non‐specific labelling by commercially‐available antibodies. We avoided this issue by using transgenic PPG‐YFP mice and took advantage of the YFP expression that occurs throughout the cytoplasm of these neurons, including their terminals (Hisadome *et al*. 2010). Immunohistochemical detection using an anti‐GFP antiserum was found to significantly enhance the native fluorescence signal of the YFP as described previously (Llewellyn‐Smith *et al*. 2011; Llewellyn‐Smith *et al*. 2013). YFP fluorescence revealed a large population of PPG‐neurons located in the granule cell layer (GCL) of the OB (Fig. 1 *A*). The somata were positioned in the upper portion of the GCL with axonal arbors reaching into the mitral cell layer (MCL) and external plexiform layer (EPL). In the GCL, the refined peroxidase labelling strategy revealed that the PPG‐neurons had stellate dendrites covered with many spines (Fig. 1 *B*) that are characteristic of deep short‐axon cells (dSACs), also named Cajal cells as described previously (Ramón y Cajal S., 1911; Price & Powell, 1970; Eyre *et al*. 2008; Eyre *et al*. 2009; Nagayama *et al*. 2014). At higher magnification (Fig. 1 *B*), we could discern that the size of the soma was slightly larger (10.20 ± 0.21 μm, *n* = 73 from three mice) compared to that reported for granule cells (Woolf *et al*. 1991; Eyre *et al*. 2009).

### GLP‐1R is expressed in the MCL and the GCL

**Figure 2:**
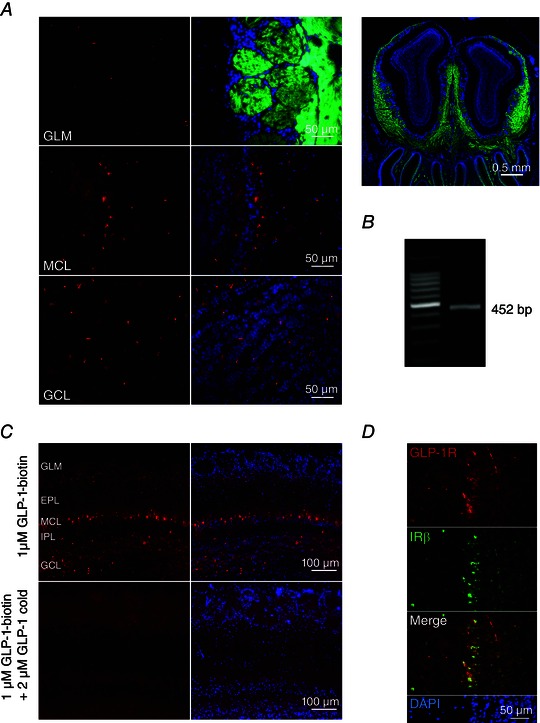
**GLP‐1R is expressed in the granular cell and MCLs of the mouse OB** *A*, left: photomicrographs of representative coronal sections of the OB of an OMP‐GFP mouse carrying a transgene for olfactory marker protein, OMP. The six‐panel composite demonstrates fluorescence labelling using an antibody directed against GLP‐1R (left; red) with no signal in the GLM, defined labelling in the MCL and scattered labelling in the GCL. Merged image on the right using double‐colour fluorescence strategy to visualize the GFP (green) and receptor (red) overlay. DAPI nuclear stain (blue). Entire OB for perspective shown on the right. *B*, RT‐PCR agarose electrophoresis gel using whole OB tissue as the template yields the anticipated size product (452 bp) for the GLP‐1R. *C*, representative photomicrograph composites as in (*A*) where a peptide binding assay was performed to visualize (top) GLP‐1 biotin conjugate binding (GLP‐1‐biotin) competing with combined (bottom) GLP‐1‐biotin plus GLP‐1 unconjugated binding (GLP‐1 cold). *D*, representative photomicrograph composite in which the section was co‐labelled with anti‐GLP‐1R (red, top), anti‐IR kinase (green, middle) with the merged image indicating MCs that putatively exhibit both co‐labelled proteins (yellow, bottom). DAPI nuclear stain (blue) is cropped below.

**Figure 3:**
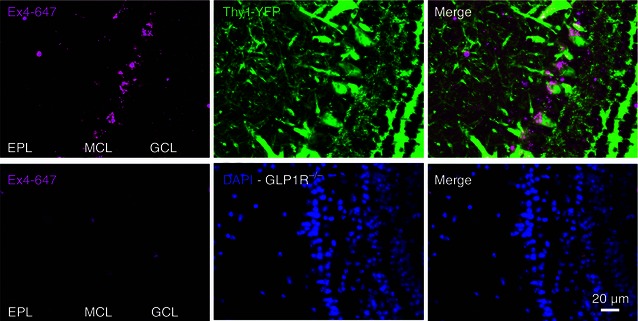
**MCs internalize of fluorescent Ex4** Photomicrograph composites of OB sections from Thy1‐YFP mice injected with fluorescent Ex4 (Ex4‐647) showing intracellular uptake of Ex4 in the MCs (top). GLP‐1R^−/−^ mice do not show uptake of Ex4‐647 in the MCL (bottom). DAPI, nuclear stain (blue); GLP‐1R^−/–,^ GLP‐1R‐null mice; Merge, merged image on the right using double‐colour fluorescence strategy to visualize the YFP (green) or DAPI (blue) with Ex4 (purple) overlay.

Following the identification of the PPG neurons in the OB, we next investigated the presence of the potential target receptor, or GLP‐1R, by immunolocalization. We used a home‐made antisera (generously provided by Scott Heller) to reveal immunoreactivity mainly in the MCL, as well as in sparse cells within the GCL (Fig. 2 *A*). Using a recently developed transgenic mouse model expressing Cre‐recombinase under the control of the GLP‐1R promoter with a ROSA26‐EYFP reporter (GLP‐1R‐Cre mice; Richards *et al*. 2014), we confirmed GLP‐1R expression in the same MCL and GCL regions (data not shown). These results are in accordance with a previous study reporting GLP‐1R mRNA in the MCL (Merchenthaler *et al*. 1999). RT‐PCR strategies produced a product of anticipated size for the receptor (452 bp) as harvested from whole OB tissue (Fig. 2 *B*). Because antisera directed against G‐protein coupled receptors, including GLP1‐R, can often create false positives, we further confirmed GLP‐1R protein expression, as well as the location, with a binding essay. Here, GLP‐1 was conjugated with biotin and detection employed streptavidin coupled to a fluorescent dye (Fig. 2 *C*). The binding assay confirmed the receptor immunolabelling, demonstrating strong binding of GLP‐1‐biotin in the MCL and GCL (Fig. 2 *C*, upper). The labelling in these layers was abolished when the OB slice was alternatively incubated in the presence of excess unbiotinylated (cold) GLP‐1 (Fig. 2 *C*, lower). It was previously demonstrated that IR kinase was expressed in the MCL (Fadool *et al*. 2000; Lacroix *et al*. 2008; Marks *et al*. 2009) and so we were interested in determining whether it might be co‐expressed with that of the GLP‐1R. Double‐colour immunofluorescence images of the OB lamina demonstrated that IR and GLP‐1R were co‐expressed in an overlapping population of MCs within the MCL (Fig. 2 *D*). Finally, as parallel support for the identity of the GLP‐1R‐expressing neurons across the MCL, we performed s.c. injection of fluorescence‐labelled Ex4 in a transgenic line, Thy1‐YFP, which expresses the fluorescent protein in MCs (Feng *et al*. 2000), as previously used to localize the distribution of the GLP‐1 receptor in the brain (Secher *et al*. 2014). Double‐colour immunofluorescence images demonstrated that all Thy1‐YFP positive MCs also reveal intracellular labelling for the fluorescent Ex4 (Fig. 3, top). Consistent with previous studies, GLP‐1 analogues are internalized in neurons after binding to the receptor (Roed *et al*. 2014; Secher *et al*. 2014; Roed *et al*. 2015). No fluorescence was observed in MCs of mice deficient for GLP‐1R (GLP‐1R^–/–^) (Fig. 3, bottom).

### GLP‐1 and Ex4 increase evoked AP firing frequency in MCs

**Figure 4:**
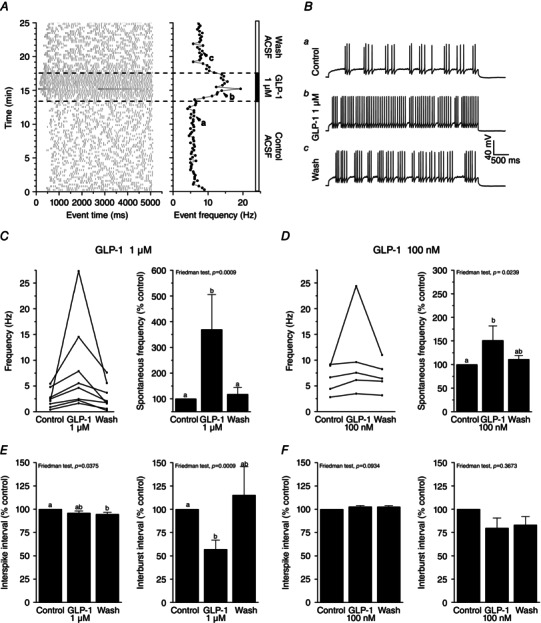
**GLP‐1 increases MC firing frequency via shortening the spike interburst interval** *A*, line graph of AP firing frequency (event frequency) and associated raster plot for a representative MC in response to bath application of 1 μm GLP‐1 to the slice. Dashed line and black bar, application of GLP‐1 proceeded by control artificial cerebral spinal fluid (Control ACSF) to establish baseline firing frequency and followed by an aCSF wash interval (Wash ACSF). *B*, enlarged resolution of AP firing pattern sampled at points *a*, *b* and *c* from (*A*). *C*, left: line graph of the AP firing frequency for the individual MCs sampled as in (*A*) and (right) bar graph of the mean firing frequency normalized to that of initial control ACSF condition. *D*, same as (*C*) but for 100 nm GLP‐1. *E*, bar graphs of the (left) ISI and (right) interburst interval for the AP activity recorded for the population of GLP‐1 responsive MCs in (*C*). *F*, same as (*E*) but for the AP activity recorded for the population of GLP‐1 responsive MCs in (*D*). *D*–*F*, different letters indicate significantly‐different means; non‐parametric repeated measure one‐way analysis of variance (ANOVA; Friedman test) followed by a Bonferoni *post hoc* comparison. *P* values are as indicated on the graphs.

To determine any functional effect of GLP‐1 on the excitability of MCs, which represent the first relay of the olfactory output to the central nervous system, we used whole‐cell, patch clamp recordings to determine the effect of bath application of GLP‐1 or its stable analogue Ex4 on the evoked AP firing frequency. Evoked APs were obtained by injecting supra‐threshold currents ranging from 25 to 100 pA. Modulation of AP firing frequency was considered significant when the addition of the peptide or analogue to the aCSF perfusion resulted in a 10% change compared to the basal frequency (aCSF only). Bath application of 1 μm GLP‐1 resulted in a rapid increase in AP firing frequency (369 ± 136%, *P* = 0.0009) in eight of the 13 cells recorded (62%), which reverted when the bath perfusion was washed with aCSF (Fig. 4 *A–C*). The effect was concentration‐dependent in that the addition of 100 nm GLP‐1 to the aCSF for 5 min significantly increased MC firing activity by 150 ± 31% (Friedman test, *P* = 0.0239) in five of the 10 recorded cells (50%) (Fig. 4 *D*). Further spike analysis determined that the change in AP firing frequency was driven by a reduction in the interburst interval rather than a change in the ISI (Fig. 4 *E* and *F*). Although GLP‐1 increased AP firing frequency, it did not change the AP shape or the RMP of MCs. In response to bath application of 1 μm GLP‐1, there was no change in the 10–90% rise time (control = 0.73 ± 0.08 ms, peptide 0.76 ± 0.09 ms; *n* = 8), width at half‐maximum amplitude (control = 1.64 ± 0.17 ms, peptide 1.63 ± 0.12 ms; *n* = 8) or RMP (control = –51.4 ± 0.8 mV, peptide –51.0 ± 1.6 mV; *n* = 8) (paired *t* test, all *P* ≥ 0.05).

**Figure 5:**
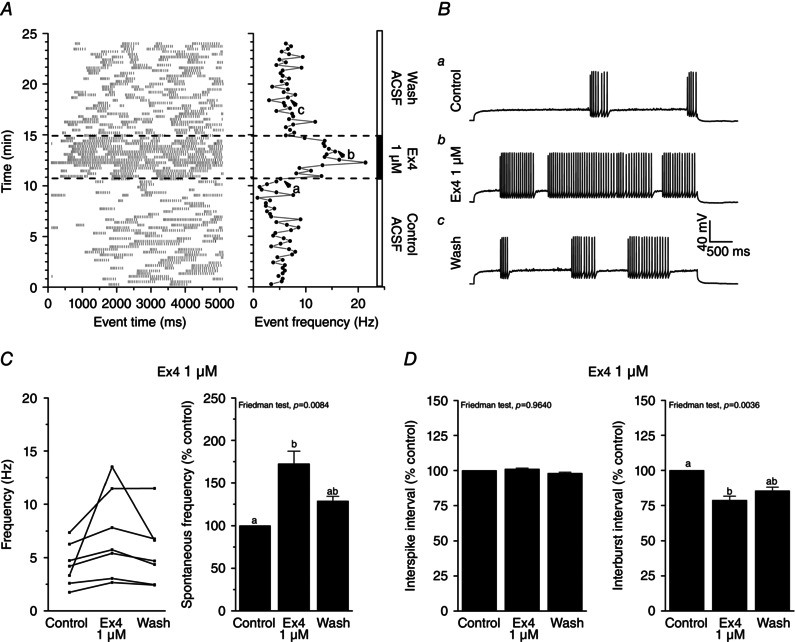
**GLP‐1 analogue, Ex4, increases MC firing frequency via shortening the spike interburst interval** *A* and *B*, same experimental paradigm as in Fig. 4 *A* and *B* but for bath application of 1 μm Ex4. *C*–*D*, same spike analysis, statistical metric and notations as in Fig. 4 *C* and *E* in the analysis of Ex4 as opposed to natural peptide GLP‐1.

Because GLP‐1 is metabolized by dipeptidyl peptidase‐IV to yield a putatively inactive metabolite [GLP‐1 (9‐36)‐amide] (Sharma *et al*. 2013), resulting in a plasma half‐life of several minutes *in vivo*, high affinity analogues (GLP‐1 mimetics) have been developed and therapeutically approved (Exenatide; Briones & Bajaj, 2006) that have indistinguishable pharmacology (Goke *et al*. 1993; Thorens & Waeber, 1993) and the ability to stimulate insulin secretion in the treatment of diabetes (Eng *et al*. 1992; Donnelly, 2012). Therefore, we applied 1 μm Ex4, a stable analogue of GLP‐1, to the slice using a paradigm identical to that for GLP‐1, which resulted in a significant increase in AP firing frequency (172 ± 15%, Friedman test, *P* = 0.0084) (Fig. 5 *A–C*) in 64% of the recorded cells (seven of 11 cells) (Fig. 5 *C*). At 100 nm, no significant change in the firing frequency was observed (*n* = 6; data not shown). Similar to GLP‐1, the observed increase in firing frequency with 1 μm Ex4 was driven by a reduction in interburst interval (Fig. 5 *D*).

**Figure 6:**
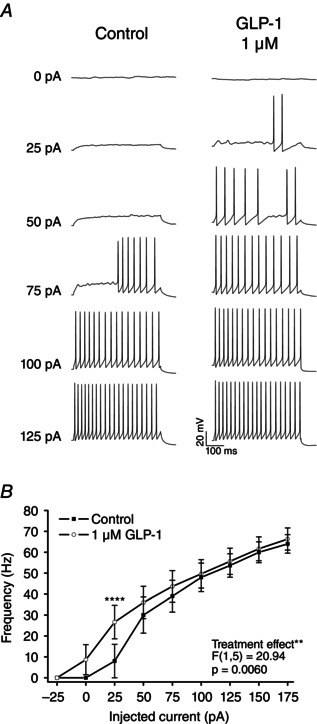
**GLP‐1 decreases the excitation threshold for MC firing** *A*, representative APs recorded in a representative MC held near resting potential and injected with a family of 500 ms long currents in 25 pA increments under control (Control) and following bath application of 1 μm GLP‐1 to the slice (GLP‐1). *B*, line graph of the mean AP firing frequency for a population of MCs (*n* = 6) recorded as in (*A*) plotted against injected current. **Significantly different means; repeated measure two‐way ANOVA; Bonferoni's *post hoc* test, using hormone treatment as the factor. **P ≤ 0.01, ****P ≤ 0.0001.

GLP‐1 might increase not only the firing frequency, but also the AP threshold to enhance MC excitability. Although the stochastic firing pattern of MCs makes the determination of a precise rheobase challenging (Balu *et al*. 2004), we were able to determine the current threshold to elicit APs before and after GLP‐1 perfusion (Fig. 6 *A*). GLP‐1 significantly decreased the excitation threshold as determined by the increased firing frequency observed across the family of injected currents that demonstrated a significant effect of peptide treatment (*F*_1,5_ = 20.94; repeated‐measures, two‐way ANOVA, *P* < 0.0060; *n* = 6). A Bonferroni's *post hoc* test showed that the difference lay with the lower current injection steps and not the higher current injections that are sensitive to changes in ISI (Fig. 6 *B*).

### Effect of GLP‐1 on the Kv1.3 potassium channel

**Figure 7:**
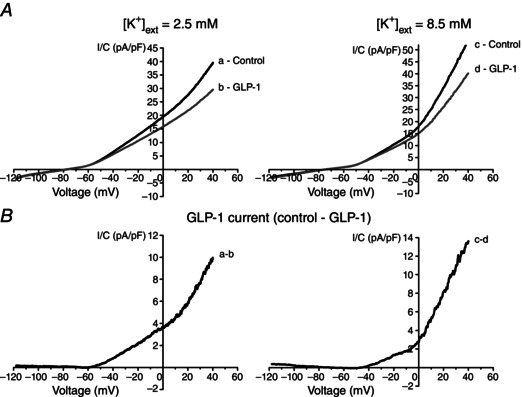
**The threshold for activation of the GLP‐1 sensitive current shifts with a change in the equilibrium potential for K^+^ (*E*_K_) generated by altering the external concentration for K^+^** *A*, representative *I*/*C* plotted relationship of currents evoked using a 400 ms voltage ramp from –120 to +40 mV before (*a*, Control, black line) and after (*b*, GLP‐1, grey line) bath application of 1 μm GLP‐1 to the slice under conditions of 2.5 mm (left) *vs*. 8.5 mm (right) external KCl concentration. *B*, the GLP‐1 sensitive current is calculated by a subtraction of *a* – *b* to yield an activation threshold of –56.3 ± 1.5 mV using a 2.5 mm external K^+^ concentration (left) compared with the right‐shifted activation threshold of –46.7 ± 3.2 mV using a 8.5 mm external K^+^ concentration (right).

**Figure 8:**
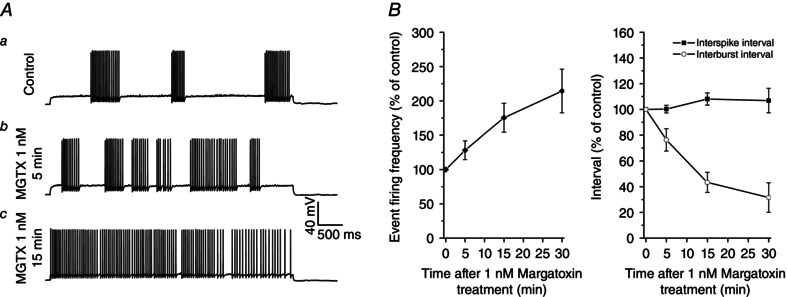
**MGTX increases AP firing frequency in MCs by decreasing the interburst interval and not the ISI** *A*, representative APs recorded in a representative MC under (*a*) baseline ASCF control bath conditions (Control), (*b*) after 5 min of 1 nm MGTX and (*c*) after 15 min of 1 nm MGTX stimulation, reflecting the slow *K*_on_ reported for this small peptide molecule that blocks the vestibule of the Kv1.3 channel. *B*, left: line graph of the mean firing frequency over time for five sampled MCs normalized to initial AP firing rate before MGTX treatment (time 0 min). Right: same population of MCs plotted to examine AP mean ISI (closed symbol) and interburst interval (open symbols) over time.

Previous studies have demonstrated that GLP‐1 or its agonists can inhibit voltage‐gated K^+^ currents such as those driven by Kv channels of the *Shaker* subfamily in pancreatic β‐cells (MacDonald *et al*. 2003; Kim *et al*. 2012; Kim *et al*. 2013) or nodose ganglion neurons (Gaisano *et al*. 2010). Kv1.3 channels are known to transition to an altered conducting state in response to changes in external K^+^ (Jager *et al*. 1998). To determine whether the GLP‐1 sensitive current was sensitive to external K^+^, we applied a 400 ms voltage ramp from –120 to 40 mV to elicit an outwardly rectifying current using two different external K^+^ concentrations (Fig. 7 *A*). This current was suppressed by application of 1 μm GLP‐1, where the difference between control and GLP‐1 conditions yielded the GLP‐1 sensitive current (Fig. 7 *B*). When using an external K^+^ concentration of 2.5 mm KCl, the activation threshold was –56.3 ± 1.5 mV. When the neurons were bathed with an external K^+^ concentration of 8.5 mm KCl, the activation threshold changed to –46.7 ± 3.2 mV (*t* test, *P* < 0.05; *n* = 5). These results indicate that the GLP‐1 sensitive current was sensitive to external K^+^, as is typical for Kv1.3 channels (Jager *et al*. 1998). Because our group previously demonstrated that Kv1.3 drives 60–80% of the potassium outward currents in MCs (Fadool & Levitan, 1998; Colley *et al*. 2004; Fadool *et al*. 2004), we examined the AP firing frequency following bath application of the American bark scorpion‐derived peptide, MGTX, a selective blocker of Kv1.3 (Knaus *et al*. 1995). As anticipated from parallel investigations (Mast & Fadool, 2012), we observed an increased AP firing frequency in MCs (Fig. 8 *A*). Interestingly, we found that this increase was correlated with a reduction of the interburst intervals and no significant change in the ISI (Fig. 8 *B*), which is similar to the effect observed after bath application of GLP‐1 and Ex4 (Fig. 4 *E* and *F* and Fig. 5 *D*).

**Figure 9:**
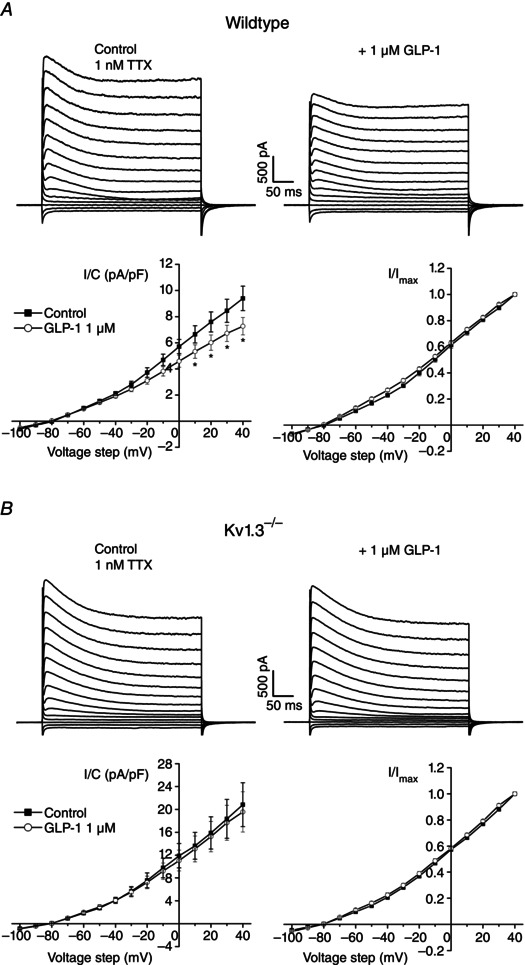
**The magnitude of MC voltage‐activated outward currents are decreased following GLP‐1 application in WT mice but not for mice with a Kv1.3‐targeted deletion (Kv1.3^–/–^)** *A*, top: representative family of voltage‐activated currents elicited by stepping the command voltage (*V*_c_) in 10 mV increments (–100 to +40 mV) from a holding voltage (*V*_h_) of –80 mV using a 400 ms pulse duration (Pd) and a 45 s interpulse interval. MC recordings were acquired from WT mice. TTX was applied to the bath to isolate outward potassium conductances (Control, 1 nm TTX) before (left traces) and after (right traces) bath application of the peptide (+ 1 μm GLP‐1). Bottom left: plotted *I*/*C* relationship for five MCs recorded as in (*A*). Solid symbols, before (Control); open symbols, after GLP‐1(GLP‐1 1 μm) bath application. Significantly different means; repeated measures two‐way ANOVA; Bonfernoni's *post hoc* test, *P ≤ 0.05; **P ≤ 0.001. Right, same as left *I*/*C* but normalized to that of the +40 mV voltage step. *B*, as in (*A*), except MCs were recorded from Kv1.3^−/−^ mice (Kv1.3^−/−^).

**Figure 10:**
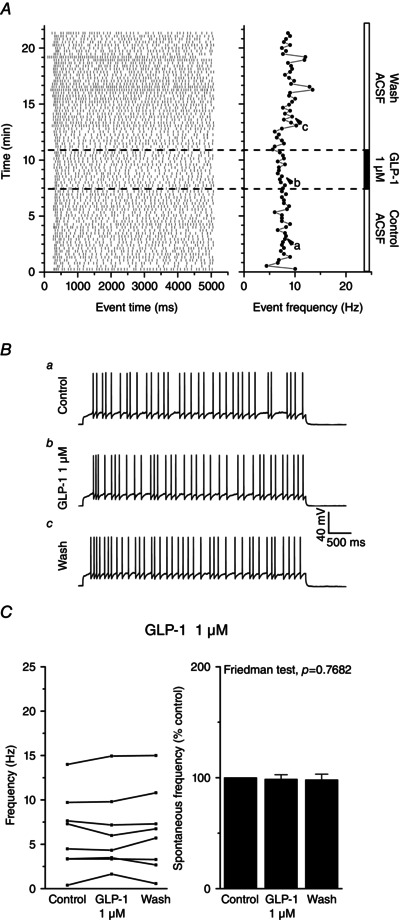
**GLP‐1 fails to modulate MC firing frequency in mice with a Kv1.3‐targed deletion (Kv1.3^−/−^)** *A* and *B*, same experimental paradigm as in Fig. 3 *A* and *B*, although MC were acquired in slices from Kv1.3^−/−^ mice. *C*, same spike analysis, statistical metric and notations as in Fig. 3 *C*, as acquired from Kv1.3^−/−^ rather than WT mice.

**Figure 11:**
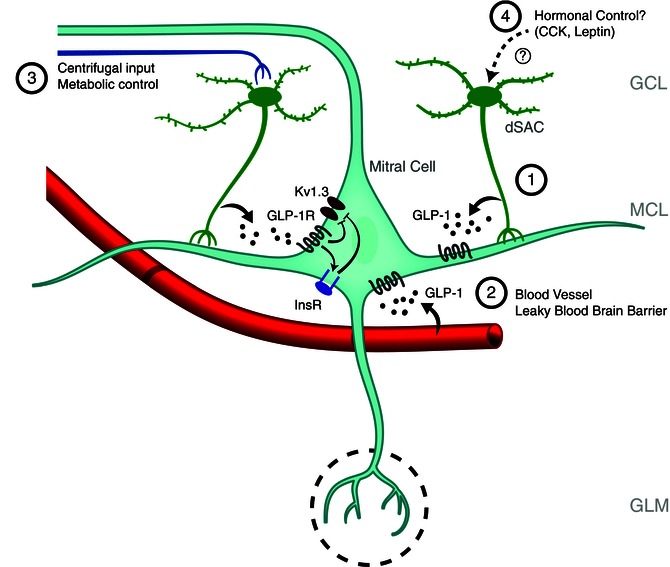
**Schematic of putative roles for GLP‐1 in the OB** Glucagon‐like peptide‐1 (GLP‐1) signalling pathways act to increase MC excitability, where several hypotheses are proposed for its role in the OB. (1) GLP‐1 could be used locally for dSAC‐to‐MC transmission or combined neurotransmission/neuromodulation of MC activity; (2) MCs could sense GLP‐1 as a metabolic signal of nutritional state derived from peripheral release in the gut and brought to the OB through the circulation; (3) centrifugal input could provide metabolic control of dSACs; or (4) hormones driven by peripheral metabolic signals may govern dSAC peptidergic transmission. Regardless of the route of GLP‐1 generation, its inducibility or regulation of release, our data demonstrate that a downstream target is the Kv1.3 ion channel. The Kv1.3 channel (brown) is predominantly expressed on MCs where it is known to be a substrate for IR kinase (blue) phosphorylation on the N‐ and C‐terminal aspects of the channel protein (Y111‐113, Y137 and Y479) (Fadool & Levitan, 1998; Fadool *et al*. 2000). Insulin‐induced phosphorylation of Kv1.3 (inhibitory line) decreases *Kv* current magnitude by decreasing the *Pr*_open_ of the channel rather than altering its unitary conductance (Fadool *et al*. 2000). GLP‐1 is known to stimulate insulin release in pancreatic β cells, a process that is glucose‐dependent, although its activation of IR kinase signalling in the brain is not known (arrow). Because Kv1.3 is part of a scaffold of protein–protein interactions (Marks & Fadool, 2007), it is highly probable that IR kinase (Fadool *et al*. 2000; Fadool *et al*. 2011), glucose signalling (Tucker *et al*. 2013) and GLP‐1 signalling converge at the level of this channel, although whether MCs homogeneously express all three metabolic factors that regulate their firing frequency is not fully understood. Deletion of the Kv1.3 channel results in a thin body type and resistance to diet and genetic obesity (Fadool *et al*. 2004; Tucker *et al*. 2008). Metabolic factors that decrease Kv1.3 ion channel activity increase the AP firing frequency in MCs that is considered to provide odour quality coding of olfactory information. Such modulation may in turn drive changes in odour‐dependent food seeking behaviours in an environment where glucose, insulin and GLP‐1 do not remain static and can also be perturbed with metabolic dysfunction or obesity. GCL, granule cell layer; MCL, mitral cell layer; GLM, glomerular layer.

To assess the effect of GLP‐1 on potassium conductance, we performed whole‐cell, voltage clamp recordings in the presence of 1 nm TTX to isolate outward potassium currents in MCs (Fig. 9). In WT animals, bath application of 1 μm GLP‐1 caused a significant suppression of outward currents at depolarizing voltage steps beyond +10 mV (seven of seven cells, repeated‐measures, two‐way ANOVA, *P* < 0.0001) (Fig. 9 *A*). *I*–*V* relationships were plotted as normalized to capacitance (*I*/*C*) to standardize any variance in MC size. No change was observed in the *I*/*I*_max_ relationship, suggesting that the decrease in conductance induced by GLP‐1 did not change the inherent voltage‐dependence of the channels underlying the outward current. Despite the outward current being driven predominantly by Kv1.3 (Fadool & Levitan, 1998), we further examined the role of this subfamily member, explicitly repeating these experiments using mice deficient for Kv1.3 (Kv1.3^−/−^). Note that the magnitude of voltage‐dependent outward potassium currents is not reduced in the absence of Kv1.3 (Control TTX traces in Fig. 9 *A vs*. *B*) as a result of the upregulation of expression of Slack channels in the Kv1.3^−/−^ mice (Lu *et al*. 2010). No change in the *I*–*V* relationships were observed when GLP‐1 was added to the aCSF perfusion for MCs prepared from these mice (eight of eight cells, *P* = 0.8089) (Fig. 9 *B*). In a parallel current clamp study (Fig. 10 *A*, *B*), GLP‐1 failed to significantly increase the AP firing frequency in Kv1.3^−/−^ animals in seven of the eight recorded cells (Fig. 10 *C*). These data strongly support a GLP‐evoked neuromodulation of MC excitability that is attributed in part to an underlying Kv1.3 conductance. A plausible model of how GLP‐1 signalling may operate in the OB is discussed below and is modelled in Fig. 11.

## Discussion

The present study is the first to report neuromodulation by incretin hormones in the olfactory system. We have used an advantageous transgenic marker to identify the PPG neurons responsible for local processing in the OB. The receptor for GLP‐1 is localized to the first‐order neurons, the MCs, which are known to be modulated by a gamut of other metabolically‐important molecules linked to the regulation of the Kv1.3 potassium channel by current suppression (Fadool *et al*. 2000; Marks *et al*. 2009; Fadool *et al*. 2011; Tucker *et al*. 2013). The GLP‐1 sensitive current is carried by a potassium conductance and modulation by the peptide is lost in Kv1.3^−/−^ mice or by pharmacological blockade of Kv1.3 channels. GLP‐1R activity in MCs is therefore well posed to alter MC excitability by modulating downstream Kv1.3 function. Moreover, if centrally acting GLP‐1 peptide in the OB is inducible, either through food availability or odour stimulation, GLP‐1R activation could trigger IR signalling, Kv1.3 phosphorylation or downstream glucose transporter translocation (Kovach *et al*. 2016) to alter energy utilization in the OB in addition to modulation of the spike firing frequency of the major output neurons.

Our combined immunocytochemical observations, biochemical binding assays and molecular data strongly support the presence of GLP‐1 secreting dSACs and the expression of the unique receptor, GLP‐1R, on MCs. The nucleus of the solitary tract, or nucleus tractus solitarii, in the brainstem and the lumbar sacral spinal cord (Llewellyn‐Smith *et al*. 2015) are the only other locations that have demonstrated cell bodies of GLP‐1 producing neurons (Larsen *et al*. 1997; Tauchi *et al*. 2008; Llewellyn‐Smith *et al*. 2011; Alhadeff *et al*. 2012; Baggio & Drucker, 2014). These studies report that GLP‐1 neurons send their axons broadly to different parts of the brain, such as the paraventricular nucleus or arcuate nucleus of the hypothalamus and the subfornical organ, whereas, in contrast, GLP‐1 neurons in the OB appear to limit their projections. The GLP‐1 secreting dSACs are consistent with the Cajal type of dSACs (see below), which have axons that reach into the MCL and EPL but do not project to higher olfactory areas outside the OB (Price & Powell, 1970; Eyre *et al*. 2008; Nagayama *et al*. 2014). Such local processing through axodendritic pathways would suggest a very local effect of GLP‐1 on neuromodulation of the olfactory output (Fig. 11, hypothesis 1), whereas the dendrites might receive centrifugal projections. Whether or not circulating GLP‐1 can reach the central nervous system remains highly controversial (Fig. 11, hypothesis 2) because the circulating GLP‐1 half‐life is short (<2 min) as a result of rapid enzymatic inactivation by DDP‐4 (Orskov *et al*. 1996; Knauf *et al*. 2005; Donath & Burcelin, 2013). Nevertheless, previous studies using radiolabelled insulin suggest that the blood–brain barrier exhibits an eight‐fold higher permeability in the OB compared to other brain regions (Banks *et al*. 1999), suggesting that circulating GLP‐1 could also play a role in olfactory modulation.

The nature of our PPG‐YFP‐positive signal in dSACs does not corroborate the earlier detection of PPG mRNA using *in situ* hybridization principally located in periglomerular cells (Merchenthaler *et al*. 1999). The PPG‐YFP‐positive signal is detected in dSACs and not superficial short axon cells surrounding the glomeruli (juxtaglomerular neurons) (Kiyokage *et al*. 2010). The dSACs are distinguished morphologically from granule cells as a result of their intermediate size between granule cells and MCs, and their cell bodies are contained in the GCL or internal plexiform layer (Eyre *et al*. 2008). With well‐impregnated Golgi sections, Ramón y Cajal (1911) and subsequent investigators distinguished seven types of non‐granule cells in the GCL, based upon soma size, shape and location; orientation of dendrites; and the presence or absence of dendritic spines, for which members of a subdivision of interneuron types were classified as dSACs (Ramón y Cajal S., 1911; Price & Powell, 1970; Schneider & Macrides, 1978). A recent review by Nagayama *et al*. (2014) provides an excellent summary of the traditional morphological and synaptic distinctions across the dSACs compared to recent reclassifications using a different nomenclature (Eyre *et al*. 2008; Eyre *et al*. 2009; Nagayama *et al*. 2014). The new classification suggests that there are three subtypes of dSACs in the inframitral layers: (1) the largest population with column‐like axonal arbors confined predominantly to the external plexiform layer, or EPL‐dSACs, and with a soma location in the GCL (also referred to as Blanes cells and Cajal cells); (2) a smaller population where the soma is retained in the internal plexiform layer with a horizontal axonal extension to the glomerular layer, or GL‐dSACs (also referred to as Horizontal cells and Golgi cells); and (3) the smallest population with axonal arbors constrained to the GCL only, or GCL‐dSAC (also referred to as Horizontal cells and Golgi cells). Our combined morphological data (Fig. 1) and biophysical evidence (Pressler *et al*. 2013; Fadool & Thiebaud, 2015) narrows down the identification of the PPG‐YFP‐positive cells to the EPL‐dSAC classification and, in particular, to Cajal cells. To our knowledge, this is the first report of a neuromodulatory effect linked to Cajal cells. Boyd *et al*. (2012) report that all dSACs are GABAergic and that Cajal cells, in particular, participate in disynaptic relays (Boyd *et al*. 2012). Understanding all of the contributions to the synaptic network and the capacity for modulation is fundamental to clarifying how Cajal cells might participate in sensory processing by controlling the main OB network via inhibition of other interneurons. Our data do not simplify the potential synaptic function of these dSACs. The present study shows that Cajal cells contain not only GABA, but also GLP‐1, and could release both to influence synaptic transmission within the OB. Moreover, GLP‐1 release from Cajal cells could be modulated by the nutritional state to fine‐tune communication between OB laminae. We also cannot exclude the possibility that locally released GLP‐1 has a solely intrinsic or paracrine neuromodulatory effect within the defined OB circuit without a metabolic‐linked function. Whether GLP‐1 levels in the OB are modified in a nutritional state‐dependent fashion is completely unknown and deserves further study.

Our mapped distribution of GLP‐1R in MCs (Fig. 2), combined with our injection of fluorescently‐conjugated Ex4 that binds selectively to GLP‐1R (Fig. 3), provides parallel support for evoked GLP‐1 release activating MCs by binding to receptors on these cells. The biotinylation results, along with the Ex4 binding assay, suggest that the receptor distribution along MCs is homogeneous. These findings are of interest given the recent studies reporting a molecular and physiological diversity of MCs that may not be a homogeneous population of neurons with respect to voltage‐gated ion channel expression (Angelo *et al*. 2012; Padmanabhan & Urban, 2014). Although our earlier immunocytochemical data (Fadool & Levitan, 1998; Fadool *et al*. 2000) support a homogeneous expression for Kv1.3 ion channel, GLP‐1R activation may have other downstream targets to mediate AP firing frequency. We also cannot exclude the possibility that there are preparation factors explaining why 50–64% of MCs exhibited enhanced firing frequency in response to GLP‐1 or its analogue. Sectioning of the lateral dendritic arbors during the slice preparation could result in less than 100% of cells being responsive in our current clamp recordings. Our electrophysiological findings show that most (but not all) of the sampled MCs exhibit a rapid and reversible increase in AP firing frequency in response to GLP‐1 or its analogue; therefore, they do not unambiguously reveal whether all MCs have identical neuromodulatory pathways. There are also a diversity of adaptor proteins interacting with K^+^ channels across the OB neurolamina (Marks & Fadool, 2007) that could refine membrane excitability and olfactory coding capacity. Finally, post‐translational modification of delayed rectifiers or their auxillary β subunits has involved GLP‐1 evoked Ser/Thr phosphorylation and acetylation (Kim *et al*. 2012). Because Kv1.3 has been well studied and is known to be a substrate for Tyr phosphorylation by several neuromodulators of MC AP firing frequency (Fadool & Levitan, 1998; Fadool *et al*. 2000; Tucker & Fadool, 2002; Colley *et al*. 2004; Fadool *et al*. 2011; Mast & Fadool, 2012), it would be intriguing to explore whether GLP‐1 stimulation of the OB can phosphorylate Kv1.3 on either Ser or Thr residues or use a cAMP independent mechanism to phosphorylate Kv1.3 on Tyr residues linked to MAPK activation (Egan *et al*. 2003).

As a post‐prandial anorexigenic signal, GLP‐1 modulation of MC excitability might be expected to be correlated with a reduction in the sensitivity to food odours. However, the change in GLP‐1 concentration at the level of the OB following a meal is unknown and an increase in MC excitability does not necessitate a gain in olfactory function. Indeed, MC intrinsic firing is complex and variable, and may promote faster odour discrimination with increased inhibition. From our earlier work (Fadool *et al*. 2000), we have demonstrated that the concentration of another neuromodulatory molecule in the OB (insulin) does not follow what is anticipated from measured levels in the plasma. For example, in these experiments, an overnight fast induced a plummeting of plasma insulin, whereas that in the OB remained high. As a post‐prandial anorexigenic signal, we do not know what explicitly activates PPG neurons, nor are we certain that an elevation of GLP‐1 in the periphery translates to a linear change centrally at the OB. Second, inhibition of MCs can be as critical to olfactory coding as excitation, and MC intrinsic firing properties can be modulated differentially to several odours, being state‐ or sensory experience‐dependent (Kato *et al*. 2012). Some intriguing work by Nunes & Kuner (2015) demonstrated that tuning MC inhibition via disinhibition of granule cells resulted in mice with faster odour discrimination without disruption of olfactory learning. Therefore the inhibitory network in the OB is working to adjust the speed of odour sensory processing. Gschwend *et al*. (2015) report that neuronal pattern separation is critical for odour discrimination learning, where enhancing the inhibition of MCs increases odour‐evoked pattern separation of activated glomeruli and improves odour discrimination learning. Lastly, Li *et al*. (2015) use a go no–go association task to reveal that changes in MC firing frequency encode information about the rewarding nature of the odour, rather than the odour identity. GLP‐1 modulation of MC firing frequency may therefore serve roles beyond that of a changed sensitivity to food odours to include how (context and circuitry) MC firing becomes changed as a critical variable influencing odour discrimination (Gschwend *et al*. 2015; Li *et al*. 2015).

Neuromodulation by GLP‐1 or its analogue has been characterized in central neurons, such as hypothalamic hypocretin/orexin neurons, or within vagal neural and motor pathways (Acuna‐Goycolea & van den Pol, 2004; Wan *et al*. 2007 *a*; Wan *et al*. 2007 *b*; Gaisano *et al*. 2010). Similar to our findings in the OB, GLP‐1 can inhibit voltage‐gated potassium channels leading to an increase in evoked Aps, such as observed in nodose ganglion neurons (Gaisano *et al*. 2010). We have previously demonstrated, both by pharmacological and genetic strategies, that Kv1.3 drives 60–80% of the outward potassium currents in the MCs (Fadool & Levitan, 1998; Colley *et al*. 2004; Fadool *et al*. 2004; Fadool *et al*. 2011); therefore, we investigated the effect of GLP‐1 stimulation in mice deficient for this *Kv* family member. Similar to that found for insulin (Fadool *et al*. 2011) and glucose (Tucker *et al*. 2013), GLP‐1 failed to decrease outward currents in voltage clamp recordings and failed to modulate AP firing frequency in current clamp experiments in which Kv1.3^–/–^ mice were substituted for WT mice. These data, combined with the fact that a Kv1.3 pore blocker selectively altered the spike train interburst but not ISI (Fig. 8), strongly support the involvement of the Kv1.3 ion channel as a downstream signalling component of GLP‐1 neuromodulation of MCs (Fig. 11). Interestingly, although all three metabolically‐important molecules affect MC firing in a Kv1.3‐dependent manner, the mechanism shows a difference in onset latency. Insulin elicits an increase in AP firing frequency that is much slower to develop (in the order of minutes) and difficult to wash out, which may be the result of the Tyr‐dependent phosphorylation observed for Kv1.3 (Fadool & Levitan, 1998; Marks *et al*. 2009; Fadool *et al*. 2011). Modulation by glucose is rapid and reversible (Tucker *et al*. 2013) and on the same time course as that for GLP‐1; however, glucose can be excitatory or inhibitory, suggesting a heterogeneity in the type of MC that exhibits glucose modulation or sensing. Although we have not yet pursued strict co‐localization of Kv1.3 and GLP‐1R, our current immunocytochemical data suggest that IR kinase and GLP‐1R are co‐localized, and our previously reported findings demonstrate that IR kinase and Kv1.3 are not only co‐localized, but also can be co‐immunoprecipitated in the presence of a variety of adaptor proteins present in the OB (Marks & Fadool, 2007; Marks *et al*. 2009). There is certainly a glucose dependence of GLP‐1 evoked insulin release. The interaction of GLP‐1 with glucose and insulin sensing pathways has been well studied in pancreatic β‐cells (Holst, 2007). One could speculate an active interaction of GLP‐1, insulin and glucose in neurons such as MCs that might co‐express GLP‐1R, IR kinase (Fadool *et al*. 2000) and glucose transporters such as sodium‐glucose transport protein‐1 or insulin sensitive, glucose transporter 4 (Al Koborssy *et al*. 2014), of which the latter is known to be regulated by Kv1.3 (Xu *et al*. 2004). For conditions during which insulin, glucose or GLP‐1 peptide were present, phosphorylation or other means of decreasing *Kv* conductance would shift glucose transporter 4 translocation to the membrane (Kovach CP, Al Koborssy D, Huang Z, Chelette B, Fadool JM, & Fadool DA, unpublished) as a positive feedback for continued glucose utilization in MCs to yield an ATP substrate for continued activation of GLP‐1R.

It is interesting that another class of chemosensory receptors has been linked to the operation of GLP‐1 signalling. The L cells of the gut have been found to express α‐gustducin, sweet taste receptors (T1R2/T1R3) and PLCβ2 (Jang *et al*. 2007), and dietary sugar has the capacity to increase sodium‐dependent glucose transporter isoform 1 in the presence of the taste transduction machinery (Margolskee *et al*. 2007). Jang *et al*. (2007) demonstrate that T1R3 and gustducin have a role in glucose‐mediated GLP‐1 release and may serve as a gut‐derived lumenal glucose sensor. Reciprocally, GLP‐1 was found to be expressed in the tongue in two distinct classes of taste cells and the receptor is found on intragemmal afferent nerve fibres (Shin *et al*. 2008). Gene‐targeted deletion of the receptor reduced sweet taste sensitivity in behaving mice as determined using brief access gustatory tests. In light of these gut/chemosensory parallels with the taste system, it is not unexpected that the olfactory system could have the ability to regulate glucose‐dependent incretin release or the ability to sense GLP‐1 hormone.

In summary, our findings generate several new hypotheses (Fig. 11) concerning the role of GLP‐1 in the OB, each of which is important to consider within the framework of future investigations: (1) GLP‐1 could be used locally for dSAC‐to‐MC transmission or combined neuromodulation of MC activity; (2) MCs could sense GLP‐1 as a metabolic signal of nutritional state derived from peripheral release in the gut and brought to the OB through the circulation; (3) centrifugal input could provide metabolic control of dSACs; or (4) hormones driven by peripheral metabolic signals may govern dSAC peptidergic transmission. Activation of the dSAC interneuron population to co‐release GLP‐1 peptide and a neurotransmitter (hypothesis 1) could be explored using select synaptic and GLP‐1 receptor inhibitors. GLP‐1 is known to be an inducible peptide and changes in the homeostatic state of the organism could be perceived through circulating GLP‐1 across a leaky blood–brain barrier at the level of the OB (hypothesis 2). If peripheral changes in GLP‐1 are detected at the OB following a meal, fasting or as a consequence of a metabolic disease state (hypothesis 2–3), or if odour signalling pathways release GLP‐1 in the OB locally (hypothesis 1), GLP‐1 sensitive potassium currents are capable of enhancing the AP firing frequency of the major output neurons or lowering their threshold for excitability. Although we cannot yet discriminate between these alternative models, the present study definitively demonstrates that the GLP‐1 signalling pathway is present in the OB and can be a regulator of neuronal activity. Taken together, the uncovered GLP‐1 signalling and the highly‐expressed glucose and insulin metabolic factors in this region are especially interesting in the context of the modulation of olfactory perception and food intake. The reported discovery of this incretin hormone in the OB challenges future investigations to examine the olfactory system as a new potential therapeutic target for controlling metabolic imbalance.

## Additional information

### Competing interests

The authors declare that they have no competing interests.

### Author contributions

NT was responsible for the collection of electrophysiological data and the receptor binding assays. IJL was responsible for immunocytochemistry in the PPG‐YFP mouse line. FG, ST and FR designed and generated the PPG‐YFP mice. NT and DF designed the experiments, assembled and interpreted the data, and wrote the manuscript. All authors shared in revising the manuscript and contributing important intellectual content. All authors have approved the final version of the manuscript and agree to be accountable for all aspects of the work. All persons designated as authors qualify for authorship, and all those who qualify for authorship are listed.

### Funding

This work was supported by NIH R01 DC013080 and DC003387 from the NIDCD, an American Heart Association (AHA) Postdoctoral Grant Award 14POST20380615, a Creative Research Council (CRC) award from FSU, a grant from the Medical Research Council, UK (MR/J013293/1) and support from the National Health and Medical Research Council of Australia, Project Grant #1025031. PPG‐YFP mice, expressing the YFP variant Venus under the control of the mouse proglucagon promoter (mGLU124 line), were generated with grant support from the Wellcome Trust.
